# Health-related quality of life assessment for patients with advanced or metastatic renal cell carcinoma treated with a tyrosine kinase inhibitor using electronic patient-reported outcomes in daily clinical practice (QUANARIE trial): study protocol

**DOI:** 10.1186/s12955-019-1085-1

**Published:** 2019-02-04

**Authors:** Guillaume Mouillet, Joëlle Fritzsch, Sophie Paget-Bailly, Astrid Pozet, Ikram Es-Saad, Aurelia Meurisse, Dewi Vernerey, Kristina Mouyabi, Diane Berthod, Franck Bonnetain, Amélie Anota, Antoine Thiery-Vuillemin

**Affiliations:** 1 Department of Medical Oncology, University Hospital of Besançon, Boulevard Fleming, F-25000 Besançon, France; 2 Methodological and Quality of Life Unit, University Hospital of Besançon, F-25000 Besançon, France; 3 INSERM, EFS BFC, UMR1098, Interactions Hôte-Greffon-Tumeur/Ingénierie Cellulaire et Génique, University Bourgogne Franche-Comté, F-25000 Besançon, France; 4 Clinical Research and Innovation Office, University Hospital of Besançon, F-25000 Besançon, France; 5 French National Platform Quality of Life and Cancer, Besançon, France

**Keywords:** Renal cell carcinoma, Health-related quality of life, Patient-reported outcome,sunitinib,pazopanib

## Abstract

**Background:**

Two main therapies, pazopanib and sunitinib, are used in the first-line setting for metastatic renal cell carcinoma (mRCC). These two tyrosine kinase inhibitors (TKI) are equally effective in terms of survival; however, they frequently induce adverse events. In this setting, Health-Related Quality of life (HRQoL) is a key element in the choice between these two treatments and the evaluation of treatment effectiveness. It could be of interest to evaluate HRQoL in daily clinical practice to aid adequate therapy choice and management. Currently, the development of information and communication technology may allow HRQoL monitoring in routine practice. The objective of the QUANARIE study is to evaluate the use of HRQoL assessment in daily clinical practice for patients with mRCC treated with TKI using electronic patient-reported outcomes (e-PRO). The present article describes the key elements of the study protocol.

**Methods:**

The QUANARIE study is an interventional, prospective, multicentre trial. Patients diagnosed with mRCC initiating sunitinib or pazopanib treatment will be invited to complete the EORTC QLQ-C30 questionnaire, nine additional questions from the EORTC items library, and the EuroQoL EQ-5D, prior to each visit with the physician. Questionnaires will be completed by patients using tablets and/or computer terminals via the e-PRO software. The physician will have real-time access to a visual summary of the HRQoL evaluation. The primary objective is to assess the proportion of patients having good compliance with Routine Electronic Monitoring of HRQoL (REMOQOL) during the first 12 months. Physicians’ satisfaction with REMOQOL will be assessed as a secondary objective. We hypothesise that 80% of patients having good compliance with REMOQOL would be meaningful. A sample size of 56 patients would be needed.

**Discussion:**

The results of this study will show whether REMOQOL is feasible on a large scale and whether patients are receptive to this new practice. This study will also determine how real-time multidimensional evaluation of patient perception can help physicians in their daily practice and how they used it in conjunction with other clinical information to manage patient care.

**Trial registration:**

ClinicalTrials.gov; Identifier: NCT03062410; First Posted: February 23, 2017; Last Update Posted: August 9, 2017.

## Background

Two main therapies, pazopanib and sunitinib, are currently used in the first-line setting for advanced or metastatic renal cell carcinoma (mRCC) [1–3]. These two tyrosine kinase inhibitors (TKI) are equally effective in terms of survival; however, they frequently induce adverse events. In the Comparz trial, a randomised phase 3 trial, non-inferiority of pazopanib to sunitinib was shown [4]. From a safety perspective, half of the patients receiving these TKI require a dose modification, and approximately 20% discontinue due to adverse events. Health-Related Quality of Life (HRQoL) scores in the pazopanib group were better than those in the sunitinib group for fatigue and treatment side effects [4]. In this setting, HRQoL is a key element in the choice between the two treatments and the evaluation of treatment effectiveness. The PISCES study showed that HRQoL and safety were key influencing factors in patient and physician preference of pazopanib or sunitinib [5].

HRQoL can be defined as the patient’s subjective perception of the impact of their disease and its treatment(s) on their daily life; physical, psychological, and social functioning and well-being. HRQoL is measured using validated self-reported questionnaires such as the EORTC QLQ-C30 cancer-specific questionnaire.

HRQoL is part of the patient reported outcomes (PROs), which includes any outcome evaluated directly by the patient, and is based on the patient’s perception of a disease and its treatment(s) [6]. It includes HRQoL, anxiety, depression, and satisfaction with cancer care, among other factors.

HRQoL at treatment start has been demonstrated to be associated with overall survival (OS) following a cancer diagnosis [7, 8], especially in renal cancer [9]. Systematic reviews have highlighted that HRQoL provides additional prognostic information compared to conventional biomedical factors; thus, PROs may better reflect patient functioning and well-being. Patients may also have a perspective regarding their functioning that is more closely related to survival than clinician perspectives (including performance status and toxicities) [10]. Moreover, HRQoL may reflect biological parameters that are not covered by other prognostic factors [11]. A relationship may also exist between better HRQoL scores and positive health behaviours [12]. Consequently, interventions that aim to improve HRQoL scores may have a positive effect on survival.

Since HRQoL captures the patient’s subjective perception of the impact of their disease and treatment, monitoring HRQoL in daily clinical practice may help to personalise patient management.

Systematic reviews have reported that routine PRO monitoring in daily clinical care, used in conjunction with other clinical parameters, is associated with an improvement in symptom control and patient satisfaction with the care received [13, 14], in addition to increased use of supportive care measures [14]. A recent randomised phase III trial showed that routine monitoring of PRO, associated with the management of alert by nurses, improves HRQoL and OS [15, 16]. More specifically, routine monitoring of HRQoL enhances communication between physicians and patients, resulting in an improvement in HRQoL [15–18]. Routine monitoring of HRQoL in daily clinical care could help to adequately choose and manage therapy, and may be of added value in the evaluation of treatment tolerance [19], customisation of supportive care [20], and the adequate assessment of treatment benefit from the patient’s point of view.

Currently, physicians mainly use OS or RECIST progression-free survival (PFS) and toxicity evaluated by the National Cancer Institute Common Terminology Criteria for Adverse Events version 4.0 (NCI CTCAE V4) as a guide to the evaluation of treatment efficiency and tolerance. Even though HRQoL is frequently evaluated in randomised phase III trials [21], routine HRQoL monitoring in daily care has not become widely implemented in routine care delivery [22]. Measuring HRQoL in routine practice has long faced a logistic issue: HRQoL questionnaires are usually filled on paper forms, and the scores must be calculated before the results can be interpreted [23, 24]. Currently, PRO measures can be collected electronically as the so-called ePRO measures, on computers, tablets, or smartphones. For instance, the software company ESD (Evaluation Software Development), has been developing the CHES Platform (Computer-Based Health Evaluation System; https://ches.pro/index.php/ches), which is a specialised software dedicated to the assessment, storage, and processing of ePRO data. One of the functions of the CHES Platform is the implementation of Routine Electronic Monitoring of HRQoL (REMOQOL) in daily oncology practice using EORTC QLQ measures. Besides, a recent systematic review reported that the QLQ-C30 was the most frequently used questionnaire in randomised controlled trials (RCTs) evaluating the routine electronic monitoring of PRO [14]. Several experiments of routine electronic monitoring of PRO have been successfully conducted [15, 18, 25]; however, it remains to be explored whether REMOQOL can be used on a larger scale, especially in oncology centres that are not necessarily involved in PRO research, without “clinician champions” [22], and in the French context of cancer-patient care.

The objective of the QUANARIE study is to evaluate the use of REMOQOL in daily clinical care for patients with mRCC treated with TKI using electronic HRQoL questionnaires and real-time feedback to physicians on a multicentre scale. This article describes key elements of the study protocol.

## Methods/design

### Study design

The QUANARIE study is an interventional, prospective, single-arm and multicentre trial involving nine French oncology centres. These nine medical institutions are located in Eastern France and cover a wide panel of centres for cancer care: three establishments specialising in cancer care (French Comprehensive Cancer Centres), three private clinics, two university hospitals, and one community hospital. The number of patients with mRCC ranges from 5 to 10 to 50–70 annually, depending on the centre*.*

The QUANARIE study will be proposed to patients diagnosed with mRCC initiating TKI anti-vascular endothelial growth factor (VEGF) treatment (sunitinib or pazopanib). The choice of TKI treatment will be at the discretion of physician, and two cohorts of equal sample size will be set up. Thus, when the required sample size is reached in one of the two cohorts, recruitment will be ceased for that cohort. There will be no randomisation or comparison between the two groups.

Patients will be invited to complete the following questionnaires prior to each visit with the physician (Table 1):

- The EORTC QLQ-C30 cancer-specific questionnaire;
- Nine items from the EORTC Item Library, exploring six supplementary symptomatic dimensions frequently described in patients treated with TKI;
- The EuroQoL EQ-5D utility questionnaire [26].

**Table 1:** Schedule of enrollment, interventions, and assessments (SPIRIT Flow Chart)

	STUDY PERIOD				
	Enrolment	First Visit	Subsequent Visit	End of Treatment	End of Study
		V1	Vx		
Informed consent	x				
Inclusion/exclusion criteria	x				
Cohort allocation (sunitinib/pazopanib group)	x				
Socio-demographic data	x				
Renal cell carcinoma history	x				
Prior medication review	x				
CHES software training	x				
Delivery of passwords, username and leaflet	x				
Level of experience with computer tools	x				
Full physical examination	x	x	x	x	
Karnofsky index	x	x	x	x	
Adverse events monitoring (NCI CTCAE V4)	x	x	x	x	
TKI prescription/dose adaptation	x	x	x	x	
Concomitant medications	x	x	x	x	
Supportive care prescription		x	x	x	
Electronic questionnaire EORTC QLQ-C30 + 9 items EORTC Library Item		x	x	x	
Electronic questionnaire EuroQoL EQ-5D-5L		x	x	x	
Need help to fill out questionnaires		x	x	x	
Location (home or hospital) where the patient completes the questionnaire		x	x	x	
Measure of time to fill the questionnaires		x	x	x	
Progression and subsequent anti-cancer treatment if applicable			x	x	x
Survival status		x	x	x	x
Satisfaction physician questionnaire					x
Satisfaction research assistant questionnaire					x

The QLQ-C30 includes 30 items and measures five functional scales (physical, role, emotional, cognitive, and social functioning), global health status (GHS), financial difficulties, and eight symptom scales (fatigue, nausea and vomiting, pain, dyspnoea, insomnia, appetite loss, constipation and diarrhoea). These scores vary from 0 (worst) to 100 (best) for the functional dimensions and GHS, and from 0 (best) to 100 (worst) for the symptom dimensions. Six supplementary dimensions: epigastralgia, mouth pain, skin toxicity, hair loss, taste changes, and bone pain, will be explored through nine items from the EORTC Item Library (http://www.eortc.be/itemlibrary/#). These items were chosen to assess the most frequent adverse events related to sunitinib and pazopanib that are not covered in the core EORTC QLC-C30 questionnaire [27].

Patients will fill out the electronic questionnaires using tablets and/or computer terminals via the CHES software) on the day of their physician visit in the waiting room or at home via a secured portal 24–48 h before.

**Fig. 1:**
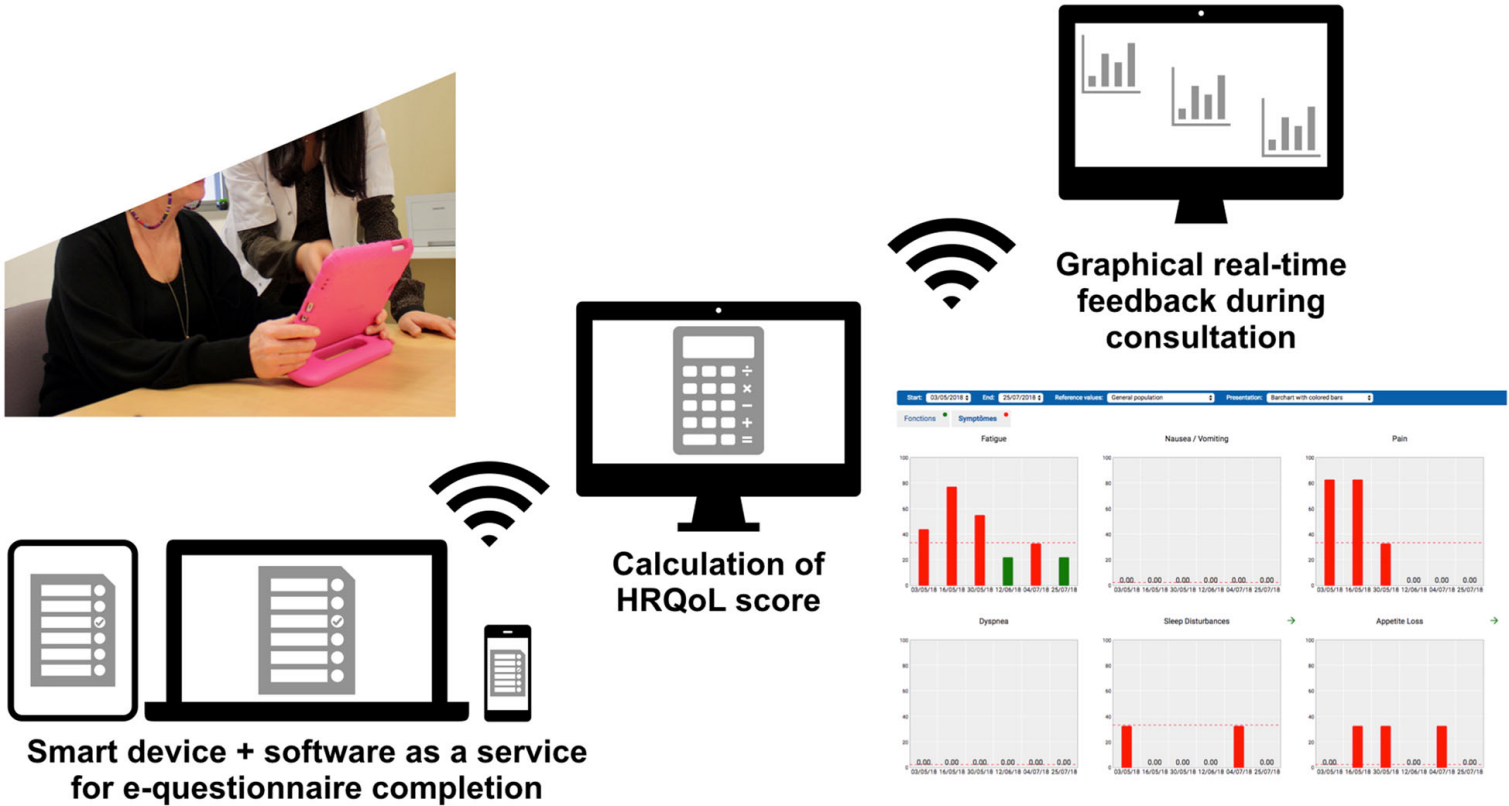
ePRO with the CHES software. Questionnaires are completed by patients using tablets and/or computer terminals via the CHES software (Computer-Based Health Evaluation System; https://ches.pro/index.php/ches) prior to each consultation at the oncology centre (waiting room) or at home via a secured portal. The physician will have real-time access to a visual summary of the HRQOL evaluation

The physician will have real-time access to a visual summary of the HRQoL evaluation (Fig. 1), which will provide the possibility of using HRQoL data during the visit to adapt the management of TKI and supportive care.

The frequencies of follow-up visits and medical exams are not pre-specified and will be as in daily clinical practice for patients treated with sunitinib or pazopanib. Investigators will have to refer to the Summary of Product Characteristics (SmPC) for the marketing authorisations of sunitinib and pazopanib for follow-up of patients and forbidden drugs.

The inclusion period is expected to last 12 months, and the follow-up duration will be two years. Final analysis of the primary objective will take place after 12 months, for a total study duration of 36 months (12 months + a 24-month follow-up).

### Description of materials

The CHES software is a service for ePRO questionnaire administration, storage of ePRO data, and immediate graphical presentation of ePRO results. Patients will be asked to complete questionnaires at each physician visit, from TKI initiation to the end of treatment. This software was developed by the academic spin-off company, ESD and is supported by the EORTC Quality of Life Group, with the aim of creating a software tool available for HRQoL studies and daily clinical practice [28].

### Characteristics of participants

#### Key eligibility criteria are the following

- Presence of histologically or cytologically confirmed renal carcinoma that is locally advanced, nonresectable, or metastatic, and initiation of an oral TKI anti-VEGF treatment (pazopanib or sunitinib);
- Estimated life expectancy greater than 3 months;
- Able to understand French and complete study questionnaires;
- Aged 18 years or older;
- Signed an informed consent form.

#### Key exclusion criteria are the following

- Prior systemic treatment for metastatic renal cancer other than immunotherapy. Pretreatment or concomitant treatment with biphosphonate or denosumab is allowed;
- Any acute or chronic disease (e.g., severe COPD) that may affect the patient’s ability to receive the treatment under study or that may render the interpretation of toxicities or adverse events difficult;
- HIV positive;
- History of active chronic hepatitis, including subjects who are carriers of the hepatitis B or C viruses;
- History of digestive pathology that could compromise the absorption of an oral TKI.

#### Key trial objectives

The primary objective of the QUANARIE study is to evaluate the proportion of patients having good compliance with REMOQOL during the first 12 months of treatment.

The key secondary objectives are the following:

- To report the number of patients to whom the study was proposed divided by the number of patients initiating sunitinib or pazopanib treatment in each centre;
- To report the number of patients who agreed to participate in this study as compared with the number of refusals divided by the total number, by centre and by molecule type (sunitinib or pazopanib);
- To estimate the rate of missing data defined as the numbers of completely or partially filled items by HRQoL questionnaire and for each measurement time. We will consider a questionnaire as filled out if at least 10 items are completed;
- To Describe the toxicities collected by the physician according to the NCI CTCAE V4 and reported by the patient according to the eight symptomatic dimensions of the QLC-C30 questionnaire and the six supplementary symptomatic dimensions;
- To evaluate Physicians’ satisfaction regarding the CHES software and REMOQOL by a questionnaire submitted at the end of the inclusion.

The exploratory objectives are the following:

- To estimate OS, defined as the time from the start of treatment to death;
- To estimate PFS, defined as the time from the start of treatment to progression or death;
- To estimate Time to deterioration of HRQoL (TTD): TTD will be defined as the time from the start of treatment to the first significant deterioration (either increase or decrease depending of the scale) in a HRQoL score as compared with the baseline score with no further significant improvement as compared to the baseline score, or death [29, 30]. Pre-specified targeted HRQoL dimensions will be global quality of life (QL), physical functioning (PF), and fatigue (FA). In these dimensions, will be considered as significant a deterioration over time of ten points at least and an improvement of eight points in QL, seven points in PF and nine points in FA [31].

### Routine electronic monitoring of HRQoL duration

Patients clinical and HRQoL data will be monitored for two years from the initiation of sunitinib or pazopanib. Patients will participate in REMOQOL until sunitinib or pazopanib is permanently discontinued due to toxicity, progression or death, and also according to a decision by the investigator, non-compliance with the protocol or loss of follow-up.

All patients included in this study will be able to leave at any time, without having to justify the reason.

After treatment discontinuation, all living patients can choose to continue REMOQOL during the subsequent therapy.

### Follow-up assessments

From treatment initiation, clinical, radiological, and biological data will be prospectively collected. Patients will fill out electronic EORTC QLQ-C30 and EuroQoL EQ-5D questionnaires at each physician visit, regardless of the interval between visits. Patients will be monitored for two years from the initiation of sunitinib or pazopanib treatment, according to the schedule of usual medical care. Following cancer progression, the physician and patient have the choice to continue REMOQOL for the subsequent therapy. Any subsequent treatment will be recorded with the date and mode of progression.

### Statistics and data analysis

#### Primary outcome definition

The primary outcome of the QUANARIE trial is the proportion of patients having good compliance with REMOQOL during the first 12 months following treatment initiation, which is defined as at least 66% of patients with filled out questionnaires during follow-up, among the overall evaluable patients.

#### Population analysis

Evaluable patients will be defined as all patients who filled out at least one questionnaire and are still alive or without progressive disease three months after the start of treatment.

#### Sample size

The following hypotheses will be tested:

H0 (null hypothesis for inefficiency): 60% or fewer patients having good compliance with REMOQOL during the first 12 months following the initiation of treatment will be considered as irrelevant;

H1 (alternative efficiency hypothesis): 80% of patients having good compliance with REMOQOL during the first 12 months following the initiation of treatment is expected.

Considering Fleming’s One-Stage design [32, 33] with a one-sided α type I error rate of 0.05 and a statistical power of 0.90, it will be necessary to include 45 evaluable patients. Due to an estimated 20% drop-out rate and non-evaluable patients, the overall number of patients included will be 56 (28 per treatment group).

#### Statistical analysis

##### Population description

Baseline variables will be described using the mean and standard deviation for continuous variables and percentages for qualitative variables. Baseline HRQoL scores will be described for each cohort.

##### Analysis of the primary endpoint

Considering the H0 and H1 hypotheses, the α and β parameters previously presented, and Fleming’s one-stage design, it will be necessary to include 45 evaluable patients in the study:

- If 32 (71%) or fewer patients are found to have good compliance with longitudinal HRQoL completion, the use of REMOQOL will be declared uninteresting in this setting;
- If at least 33 (73%) patients are found to have good compliance with longitudinal HRQoL completion, the use of REMOQOL will be declared interesting.

The probability of concluding efficacy, when *p* = 60.0%, is α = 0.045%.

The probability of concluding inefficacy, when *p* = 80.0%, is β = 9.9%.

##### Analysis of secondary endpoints

Secondary endpoints will be described using the mean and standard deviation for continuous variables and percentages for qualitative variables. The rate of missing data at each HRQoL measurement time will be reported. HRQoL results within treatment groups and in all evaluable patients will be assessed using descriptive statistics. The adverse events occurring during the study, according to the NCI CTCAE V4, will be reported by group. There will be no comparative analysis between the sunitinib and pazopanib groups. Survival outcome (OS, PFS, TTD) will be estimated using the Kaplan–Meier method. Median survival will be described with confidence intervals (one-sided statistical test for significance (α: 5%)) in each of the treatment groups and in all evaluable patients.

## Discussion

A better understanding of the pathogenesis of metastatic clear-cell renal-cell carcinoma has made it possible for the development of novel therapeutics capable of changing patient management [34]. Some tyrosine kinase receptors and their ligands have been shown to play an important role in tumour growth and angiogenesis. Inhibition of VEGF signalling through the use of antibodies or VEGF antagonists has demonstrated potent antitumour activity in the mRCC setting [35]. To date, nine targeted therapies have been approved by regulatory authorities in the United States and Europe, which have revolutionised the treatment of mRCC and largely contributed to what can be called the chronic care of this disease. As an example, in an unselected “real-life” cohort of 224 metastatic patients, the median OS for patients with a good prognosis, according to the Heng prognostic score [36], was 32 months [34]. This allowed expansion of the number of treatment options and sequences in this setting. Moreover, new generation immunotherapy has recently been approved by the FDA for a first-line setting [37].

From the patient’s perspective, it is essential that this improvement in the duration of survival is not at the cost of high toxicity; therefore, improvement of TKI tolerance has become a major topic of research in the mRCC field. For instance, the PISCES study evaluated patient preference of sunitinib or pazopanib [5]. Some trials have evaluated alternative administration schedules of sunitinib to improve its tolerability [38, 39]. In daily clinical care, the management of side effects related to TKI is an important part of mRCC patient care to enhance treatment compliance. A German group highlighted the fact that the benefit of these drugs may differ in routine practice as compared with patients included in clinical trials [40]. In parallel, there is increasing evidence that PRO in routine care may help in the management of sides effects [15, 16].

Several studies have provided evidence that the regular use of PROs, including HRQoL, improves communication between the patient and physician, in addition to HRQoL scores and survival [14]; however, these studies often took place in expert centres. The QUANARIE study will provide an opportunity to evaluate whether routine HRQoL monitoring is feasible outside expert centres*.* Furthermore, most of these trials have focussed on the management of side effects with PROs that only evaluate the occurrence of adverse events and their severity. In the QUANARIE study, the evaluation of HRQoL with the EORTC QLQ-C30 allows a multidimensional evaluation of the patient’s point of view, using functional and symptom scales. While EORTC QLQ-C30 is the most frequently used questionnaire in routine PRO, it is not the most frequent tool used in mRCC RCTs [21]; however, the items evaluated by the EORTC QLQ-C-030 and the National Comprehensive Cancer Network - Functional Assessment of Cancer Therapy-Kidney Symptom Index 19 (NCCN-FACT FKSI-19) are relatively similar [41], with both exploring symptoms such as asthenia, pain, nausea, diarrhea, and dyspnea, in addition to “function and well-being”. Nevertheless, while FKSI questionnaires are the most used in mRCC RCTs, they do not cover most symptoms related to TKI.

In the COMPARZ trials, a specific questionnaire, the Hand-Foot and Mucositis Symptom and Impact Questionnaire (HAMSIQ) was used in addition to the FKSI 19 questionnaire for the assessment of mouth/throat, hand/foot soreness symptoms, and subsequent limitations in patients receiving pazopanib or sunitinib for mRCC [27, 42].

Since the HAMSIQ questionnaire is not freely available, we used the EORTC Items Library to create new questionnaires based on the database of items used in fully and partially validated EORTC Quality of Life questionnaires. We added supplementary items to the QLQ C30 questionnaire that target the most frequent adverse events reported in sunitinib and pazopanib trials.

The integration of HRQoL assessment into daily clinical care faces multiple barriers such as material and IT limitations and implementation of an intervention that may increase the workload of already busy physicians or a training physician who is not familiar with interpreting HRQoL measures [23, 43–46]. The QUANARIE trial is part of a larger project led by the Methodological and Quality of Life Unit in Oncology (UMQVC) with the aim of integrating HRQoL monitoring as a complementary tool into daily clinical practice. Two other studies are currently recruiting patients: the GYNEQOL-Pilot [NCT02864797] and the QOLIBRY trial [NCT02844608]. To date, more than 360 patients treated for different types of cancer (gynecological, colorectal, lung, breast, and kidney) with different types of treatment (chemotherapy, TKI, and immunotherapy) have been included in these three clinical trials. The results of the QUANARIE study will demonstrate whether REMOQOL is feasible on a large scale and whether patients are receptive to this new practice. This study will also show how real-time multidimensional evaluation of patient perception can help physicians in their daily practice and how they can use it in conjunction with other clinical information to manage patient care.

## Abbreviations

CHES: Computer-Based Health Evaluation System software
COPD: chronic obstructive pulmonary disease
EORTC: European Organisation of Research and Treatment of Cancer
ePRO: electronic patient-reported outcome
EQ-5D: European Quality of Life – 5 Dimensions Questionnaire
ESD: Evaluation Software Development
FA: Fatigue
FDA: US Food and Drug Administration
HRQoL: Health-Related Quality of Life
ORR: Overall response rate
OS: Overall survival
PF: Physical Functioning
PFS: Progression-free survival
PRO: Patient-reported outcome
QL: Global Quality of Life
QLQ-C30: Quality of Life Core Questionnaire
RCC: Renal cell carcinoma
REMOQOL: Routine Electronic Monitoring of HRQoL
SmPC: Summary of product characteristics
TKI: Tyrosine kinase inhibitors
TTD: Time to HRQoL deterioration
TTP: Time to progression
VEGF: Vascular endothelial growth factor
